# Preoperative hemoglobin thresholds for survival equity in women and men

**DOI:** 10.3389/fmed.2024.1334773

**Published:** 2024-03-13

**Authors:** Florian Rumpf, Lotta Hof, Oliver Old, Patrick Friederich, Jens Friedrich, Josef Thoma, Maria Wittmann, Kai Zacharowski, Suma Choorapoikayil, Patrick Meybohm, German Patient Blood Management Network Collaborators, Olaf Baumhove

**Affiliations:** ^1^University Hospital Würzburg, Department of Anaesthesiology, Intensive Care, Emergency and Pain Medicine, Würzburg, Germany; ^2^Department of Anaesthesiology, Intensive Care Medicine and Pain Therapy, University Hospital Frankfurt, Goethe University Frankfurt, Frankfurt, Germany; ^3^Department of Anaesthesiology, Operative Intensive Care Medicine and Pain Therapy, Muenchen Klinik Bogenhausen, Munich, Germany; ^4^Department of Anaesthesiology and Intensive Care Medicine, Klinikum Leverkusen, Leverkusen, Germany; ^5^Department of Anaesthesiology and Operative Intensive Care Medicine, Ortenau Klinikum, Offenburg-Kehl, Germany; ^6^Department of Anaesthesiology and Operative Intensive Care Medicine, University Hospital Bonn, Bonn, Germany

**Keywords:** hemoglobin, equitable health care, anemia, perioperative management, perioperative medicine

## Abstract

Anemia affects humans throughout life, and is linked to higher morbidity and mortality. Unclear is whether hemoglobin values are equivalent between women and men. This study evaluates the association of preoperative hemoglobin levels with in-hospital mortality and estimates thresholds for survival equity between men and women. All adult patients undergoing surgery between 2010 and 2019 from 14 German hospitals were included in the study. Thresholds for survival equity were determined with generalized additive models. In total, 842,130 patients with a median in-hospital follow-up time of 7 days were analyzed. During follow-up 20,370 deaths occurred. Preoperative hemoglobin stratified in-hospital mortality (log-rank test *p* < 0.001) and was associated with mortality independently of demographic risk, surgical risk and health status. For each 1 g/dL reduction in preoperative hemoglobin, the odds of mortality increased by a factor of 1.22 (95% CI 1.21–1.23, *p* < 0.001). A preoperative hemoglobin threshold of 10.5 g/dL reflected equivalent risk for both male and female patients. Hemoglobin levels below 10.5 g/dL had higher risk of mortality for women than for men. The findings from this study aid evidence-based thresholds, inform anemia management and promote equitable care, thus enhancing patient outcomes.

## Highlights

- This study confirms the strong relationship between anemia and in-hospital mortality.
- A hemoglobin threshold of 10.5 g/dL posed equal mortality risk for male and female patients, with levels below it elevating risk more for women.

## 1 Introduction

Anemia is a common health issue that can affect people of all ages and is linked to higher rates of illness and death. The World Health Organization (WHO) has emphasized that appropriate subgroup thresholding would improve the diagnosis and management of anemia (1). The anemia definition by the WHO involves hemoglobin (Hb) levels < 13 g/dL for men, <12 g/dL for non-pregnant women, and < 11 g/dL for pregnant women (2, 3). These values reflect the lower end of the distribution of Hb values in healthy patients. However, establishing Hb thresholds that are applicable to different populations and clinical settings remains a challenge (4, 5). So far, there is no anemia definition, which is outcome-related.

An especially vulnerable population for anemia are patients undergoing surgery. Preoperative anemia has been associated with postoperative adverse outcomes in both cardiac and non-cardiac surgery (6, 7). Following Patient Blood Management recommendations, the prevalence of anemia should be reduced before surgical interventions (8, 9). Women often have lower Hb values and are thus considered to have a higher tolerance to low Hb values (10, 11). In the surgical setting women additionally have a considerable disadvantage due to lower blood volume. Both factors could contribute to adverse outcomes for women undergoing surgery. It has been suggested that women have higher transfusion rates, complication rate and mortality compared to men undergoing similar surgical procedures (12–14). The question therefore remains what Hb values are equivalent between women and men in respect to mortality risk.

The German Patient Blood Management Network provides a unique opportunity to evaluate preoperative Hb thresholds across a broad population of surgical patients (15). Access to data from more than 1 million patients enables data-driven estimation of outcome-based Hb thresholds. This study models the variation in Hb thresholds across the life cycle, evaluates the association of preoperative Hb levels with in-hospital mortality and estimates thresholds for survival equity between men and women.

## 2 Methods

### 2.1 Study population

This study included all patients aged ≥18 years who underwent non-obstetric surgery, were discharged from 14 German hospitals between January 1st, 2010, and December 31st, 2019, and had accessible preoperative Hb levels. Detailed methods have been published previously (15). Briefly, data were individually gathered from the electronic systems of participating hospitals, and supplemented with pharmacy and blood bank data by the local IT staff. To ensure privacy, anonymization was performed before aggregation at the data center. Routine error checks and validation were done by center-specific experts and PBM network biostatisticians. A healthy subpopulation was defined as patients with a Charlson comorbidity score of 2 or less undergoing minor surgery.

The study received approval from the ethics committee of the University Hospital Frankfurt (Ref. 318/17), as well as the ethics committees of all participating centers. Additionally, the Hessian data protection officer granted approval (Ref. 43.60; 60.01.21-ga, 24 October 2018). The ethics committee waived the requirement for written informed consent from patients.

### 2.2 Statistics

The primary outcome of this study was in-hospital mortality and secondary outcomes were transfusion rate and length of hospital stay. In all steps of the analysis generalized additive models with linear, nonlinear and random effects were used (16). The 5th percentile of preoperative Hb values was estimated for increasing age with additive quantile regression (17). Univariate comparison between men and women was done with Pearson’s chi-squared test for counts and Kruskal–Wallis rank sum test for numeric variables.

For multivariate modeling candidate variables were selected by acyclic graphing based on literature and expert consensus. The variables included in the final models were then refined with forward selection. To capture demographic changes across a decade of surgical interventions, mortality rates specific for age, time of intervention and sex were calculated based on data from the German Federal Bureau of Statistics (18). All mortality models were adjusted for this demographic risk. To capture the health status of patients, models were also adjusted for the Charlson comorbidity index (19). Additionally, models were adjusted for age, sex, surgical risk and length of stay as fixed effects and treating hospital as a random effect. Surgical risk assessment was based on the performed procedures. Surgical procedure codes were ranked by the associated mortality risk yielding distinct risk categories. This was combined with an expert classification into minor and major surgery based on Supplementary Table S1. Both measures were jointly evaluated to capture the *a priori* risk associated with a surgical procedure.

A robust technique for estimating lower limits of normal are outcome-based methods that estimate values that are associated with a higher risk of adverse outcomes (20). To investigate what Hb values are equivalent between women and men with regard to mortality, interactions between Hb thresholds and sex were examined. Models with a threshold difference between 2.5 and −1.5 g/dL Hb between men and women were estimated continuously. Survival equity was defined as the Hb thresholds with the smallest interaction with sex.

## 3 Results

**Figure 1:**
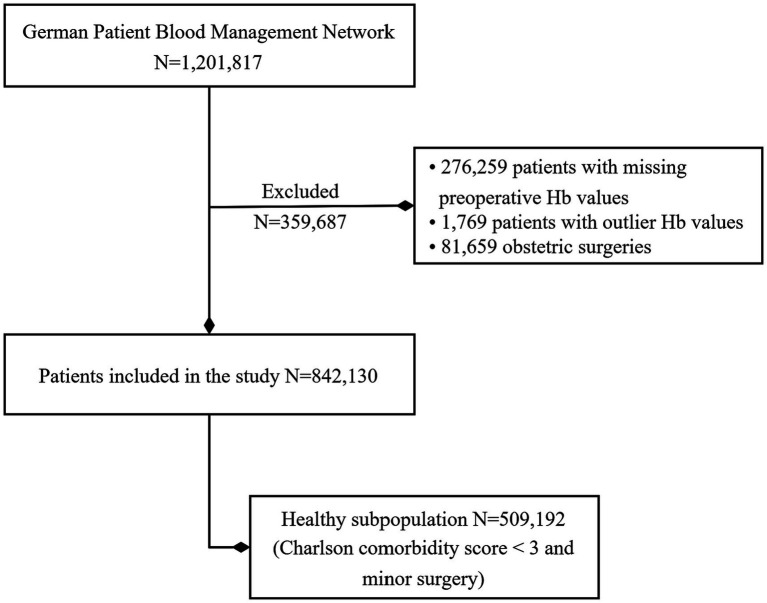
Study population: patients included in this study from the German Patient Blood Management Network.

A decade of surgeries from 14 hospitals led to evaluate 1,201,817 patients for this study. A broad range of surgeries was included to capture a diverse surgical population. Most frequent were surgeries on the Visceral surgery 19.7%, Trauma and orthopaedic surgery 16.5%, Urology 9.6%, Neurosurgery 8.4%, and Cardiac surgery 7.7%. Patients with missing preoperative Hb values (*n* = 276,259), outlier Hb values outside the range 4–18 g/dL (*n* = 1,769), and obstetric surgeries (*n* = 81,659) were excluded. 842,130 patients were included in this study (Figure 1). During a median in-hospital follow-up time of 7 days (IQR 9), in-hospital mortality was observed for 20,370 patients. Cohort characteristics are given in Table 1. The clinical features of women and men undergoing surgery exhibit distinct differences.

**Table 1:** Cohort characteristics for patients included in this study.

	Male (*N* = 456,356)	Female (*N* = 385,774)	*p*
Age (years)	64 (51, 75)	63 (48, 76)	<0.001^a^
Charlson comorbidity index	0.0 (0.0, 2.0)	0.0 (0.0, 2.0)	<0.001^a^
Surgical discipline			<0.001^b^
Visceral surgery	89,415 (19.6%)	76,529 (19.8%)	
Trauma and orthopaedic surgery	67,775 (14.9%)	71,463 (18.5%)	
Urology	62,580 (13.7%)	18,597 (4.8%)	
Neurosurgery	37,278 (8.2%)	33,327 (8.6%)	
Cardiac surgery	43,161 (9.5%)	21,512 (5.6%)	
Vascular surgery	35,543 (7.8%)	23,979 (6.2%)	
Dermatology	26,422 (5.8%)	20,652 (5.4%)	
Ophthalmology	20,764 (4.5%)	19,683 (5.1%)	
Otorhinolaryngology	24,326 (5.3%)	15,536 (4.0%)	
Gynaecology	348 (0.1%)	35,917 (9.3%)	
Oral-maxillofacial surgery	19,594 (4.3%)	15,152 (3.9%)	
Surgery of the hematopoietic- and lymphatic system	8,639 (1.9%)	15,263 (4.0%)	
Thoracic surgery	14,517 (3.2%)	8,645 (2.2%)	
Endocrine surgery	3,397 (0.7%)	7,922 (2.1%)	
Other surgeries	2,597 (0.6%)	1,597 (0.4%)	
Preoperative hemoglobin (g/dL)	14.0 (12.5, 15.1)	13.0 (11.8, 13.9)	<0.001^a^
Postoperative hemoglobin (g/dL)	12.3 (10.3, 14.1)	11.7 (10.2, 13.0)	<0.001^a^
Perioperative hemoglobin change (g/dL)	−0.8 (−2.3, 0.0)	−0.7 (−1.9, 0.0)	<0.001^a^
Red cells (any)	59,224 (13.0%)	47,172 (12.2%)	<0.001^b^
Platelets (any)	19,130 (4.2%)	9,456 (2.5%)	<0.001^b^
Plasma (any)	16,286 (3.6%)	9,180 (2.4%)	<0.001^b^
Fibrinogen (any)	10,897 (2.4%)	5,375 (1.4%)	<0.001^b^
Prothrombin complex concentrate (any)	17,225 (3.8%)	8,537 (2.2%)	<0.001^b^
Length of hospitalization (days)	7 (4, 13)	7 (4, 12)	<0.001^a^

The table gives median and interquartile range (IQR) for continuous variables and counts for categorical variables. For blood products the count reflects the number of patients that received any blood product.

a Kruskal–Wallis rank sum test.

b Pearson’s chi-squared test.

**Figure 2:**
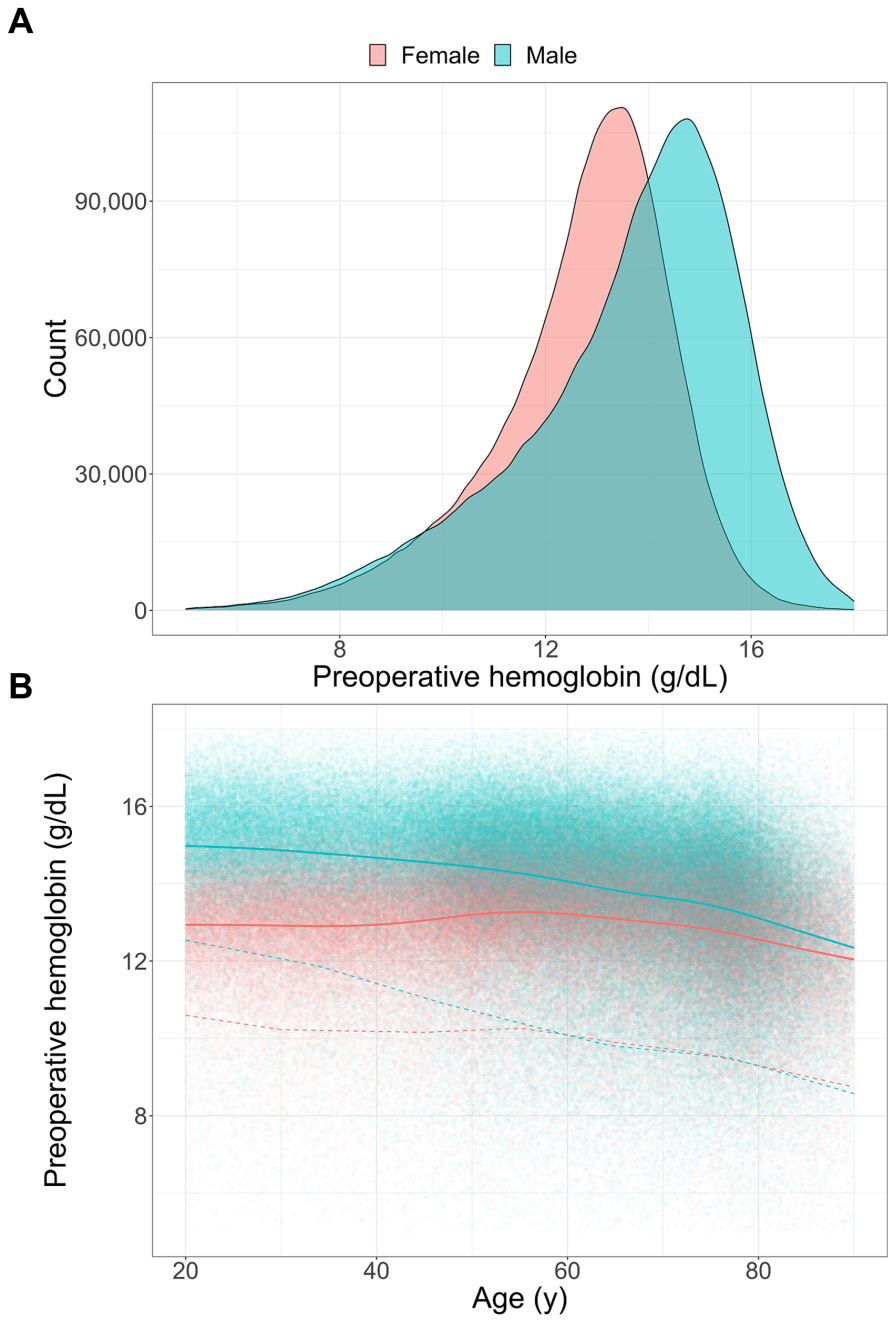
Preoperative hemoglobin levels: **(A)** distribution of preoperative hemoglobin in female (*n* = 385,774) and male patients (*n* = 456,356), **(B)** smoothed conditional means (solid line) with the 5th percentile (dashed line) for a healthy subpopulation (Charlson comorbidity score <3, minor surgery). Measures are given continuously for age in female (*n* = 234,430) and male patients (*n* = 274,762).

While preoperative Hb levels were normally distributed for both male (*n* = 456,356) and female patients (*n* = 385,774), male preoperative Hb levels followed a more left skewed distribution (Figure 2A). The median preoperative Hb level in male patients was 1 g/dL higher than in female patients (14.0 g/dL in men versus 13.0 g/dL in women). For both women and men preoperative hemoglobin below WHO thresholds were associated with an increase of red cell transfusions. For hemoglobin values below 15 g/dL men received increasingly more red cell transfusions than women (Supplementary Figure S1).

Preoperative Hb levels did not only vary between men and female patients but also decreased significantly with age in a healthy subpopulation. While healthy patients under 40 years old had a mean preoperative Hb value of 15 g/dL for men and 13 g/dL for female patients, this difference narrowed with increasing age (Figure 2B). Interestingly, this is mostly due to a decrease of mean Hb of up to 3 g/dL in male patients over 40 years old compared to patients under 40 years old. In female patients the mean Hb rises at the age of 40 and then decreases slightly in older patients. The lowest 5% of healthy patients undergoing surgery had a similar difference in preoperative Hb levels under 40 years of age but this difference decreased even more dramatically with age and after the age of 55 the lowest 5% female and male patients had almost identical preoperative Hb values (Figure 2B).

**Figure 3:**
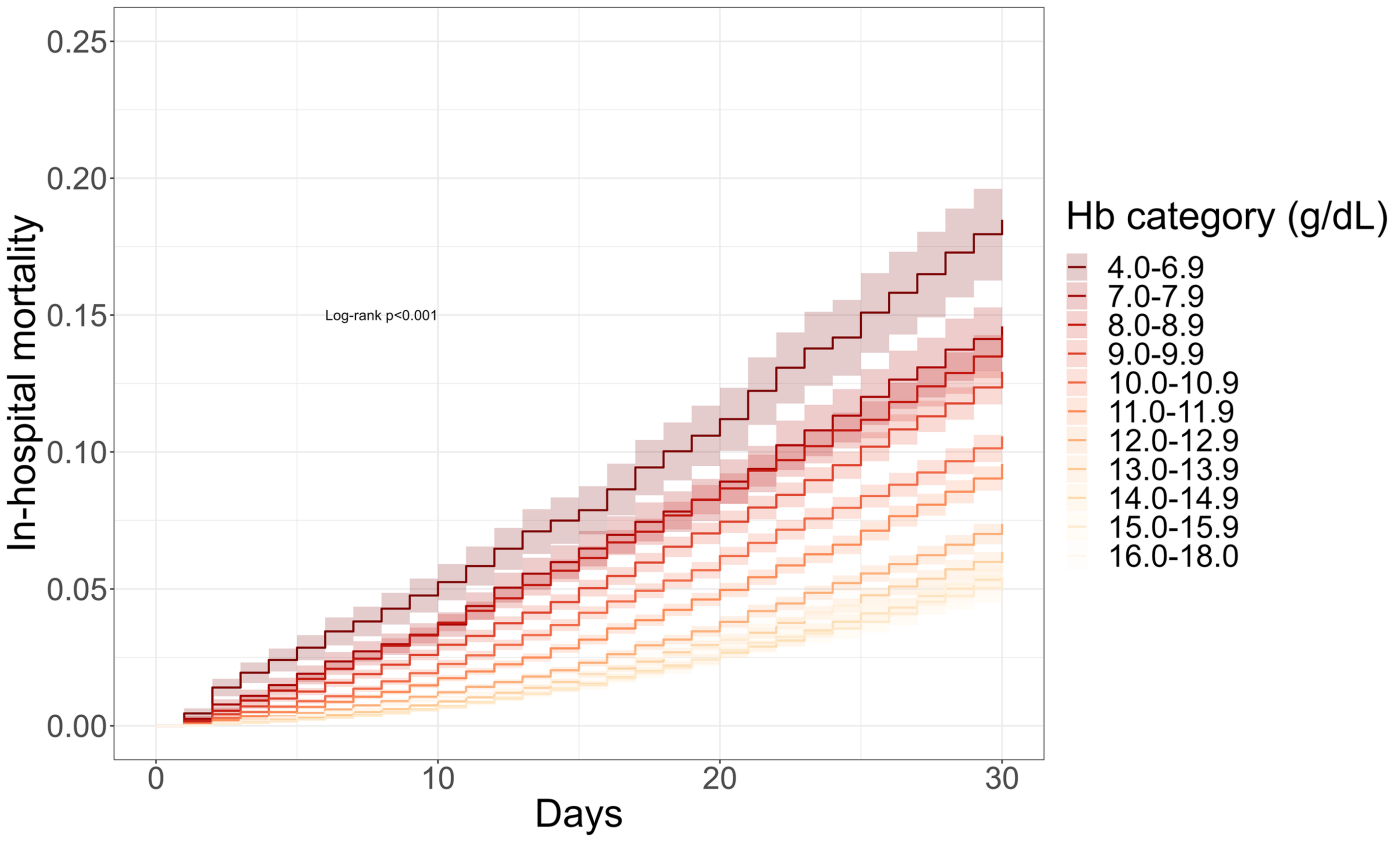
Preoperative hemoglobin stratifies in-hospital mortality: survival curves were derived from the Kaplan–Meier estimator stratified by preoperative hemoglobin. Decreasing hemoglobin levels were associated with increasing mortality risk (log-rank *p* < 0.001).

Decreasing preoperative Hb levels were associated with increased death rates (Supplementary Table S2) and were able to stratify in-hospital mortality (Figure 3, log-rank test *p* < 0.001). Notably, the association of preoperative Hb with mortality remained significant even after accounting for various factors such as health status, demographic risk and type of surgery. A decrease of 1 g/dL in preoperative Hb was associated with a 1.20 OR (95% CI 1.19–1.21, *p* < 0.001) for an increased risk of mortality (Supplementary Table S3). This association was also significant for every surgical discipline in subgroup analysis except for endocrine surgery. In the cardiac surgery subgroup preoperative hemoglobin had the lowest OR per 1 g/dL decrease (OR 1.11, 95% CI 1.09–1.13, *p* < 0.001), while the oral-maxillofacial surgery subgroup had the highest OR per 1 g/dL decrease (OR 1.30, 95% CI 1.28–1.32, *p* < 0.001). Lower preoperative Hb values increased red cell transfusion requirements and prolonged hospital stay. Every 1 g/dL drop in preoperative Hb had a 1.72 OR (95% CI 1.71–1.73, *p* < 0.001) for any red cell transfusion. Likewise, every 1 g/dL drop in preoperative Hb prolonged the length of hospital stay by 0.58 days (95% CI 0.56–0.60, *p* < 0.001).

**Figure 4:**
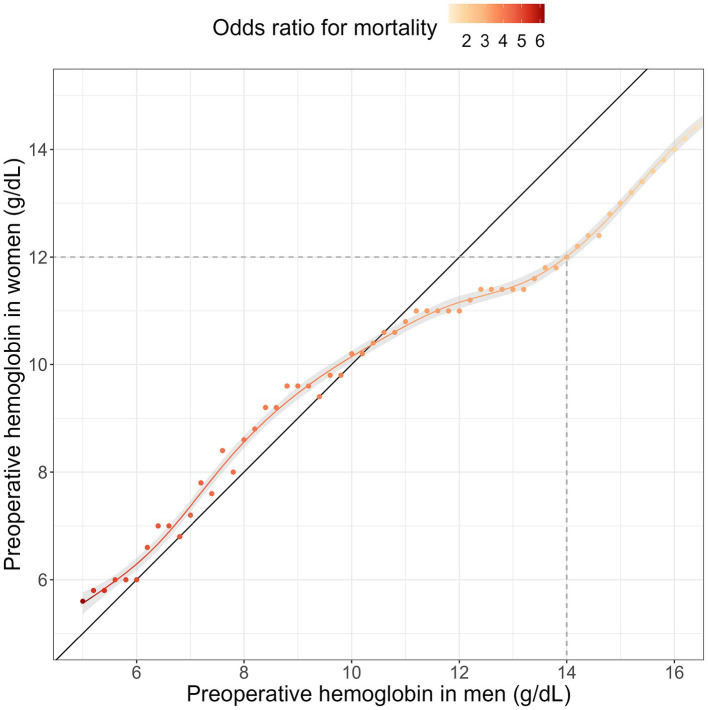
Hemoglobin thresholds for survival equity between women and men: in order to investigate comparable hemoglobin levels linked to mortality in both men and women, interactions of hemoglobin thresholds and sex were analyzed using generalized additive models. Survival equity was defined as the hemoglobin thresholds that show the least difference based on sex. The smooth line indicates similar hemoglobin levels for both genders. The color scale illustrates the associated risk (odds ratio) with these thresholds.

To determine equitable Hb values for risk of mortality, we estimated thresholds considering health status, demographic risk, and type of surgery for men and women. Figure 4 gives preoperative Hb values with equivalent mortality risk between women and men. Hemoglobin thresholds that show the least difference based on sex are illustrated as a solid line. If preoperative Hb values between women and men were to be equivalent 1:1, Hb thresholds could be represented by the identity function (Figure 4, solid black line). At the upper end of the spectrum Hb thresholds for survival equity were estimated 2 g/dL higher for men than for women. That means for women a threshold of 12 g/dL was equivalent in mortality to a threshold of 14 g/dL in men (Figure 4, dashed grey line). Notably a threshold of 10.5 g/dL was associated with equivalent mortality for both male and female patients. At this threshold women received 1.4 red blood cell units on average while men received 2.0 units (Supplementary Figure S1). Lastly, at the lower end of the spectrum survival equity was estimated 0.5 g/dL higher for women than for men. That means for women a threshold of 8 g/dL was equivalent in mortality to a threshold of 7.5 g/dL in men (Figure 4).

## 4 Discussion

The results presented in this study quantify the impact of low Hb levels on in-hospital mortality and their differential impact on women and men. A Hb threshold of 10.5 g/dL posed equal mortality risk for male and female patients, with levels below it elevating risk more for women. Furthermore, low preoperative Hb values increased in-hospital mortality, transfusion requirements and length of hospital stay.

The observed difference in Hb levels between male and female patients emphasizes the importance of sex-specific thresholds to ensure optimal clinical outcomes. Sex-specific differences in anemia have only recently been warranted in the Anaemia Global Burden of Disease Study (5). This falls in line with the WHO call for subgroup thresholding in anemia management (1). The identification of sex-specific thresholds for survival equity adds valuable insights to the management of anemia and offer clinicians practical guidance for assessing anemia severity. The highlighted sex-specific differences also align with findings from previous studies (14, 21–23). The increased risk of mortality in women compared to men with Hb levels below 10.5 g/dL, indicates that women did not have a higher tolerance to low Hb values. This finding differs from previous studies that have suggested a higher tolerance for low Hb values in women (10, 11). These differences can be explained in part by the incomplete adjustment for confounders in these retrospective studies. This phenomenon is also comprehensible in our study through an examination of the adjusted and unadjusted associations. Although men exhibited a higher mortality rate at low hemoglobin levels univariately (Supplementary Table S2), after accounting for confounding variables such as health status, surgical risk, demographic factors, and blood transfusions, women were found to be at a greater risk at lower hemoglobin values (Figure 4). The significance of these confounding variables is underscored in Table 1, which illustrates the distinct clinical traits between men and women. Men typically exhibit higher morbidity and mortality rates compared to women in the same age group. Therefore, overlooking sex-specific characteristics, such as demographic mortality risks, could introduce biases when comparing Hb values between men and women. From an evolutionary perspective women could be expected to have a higher tolerance to a drop in Hb due to habituated blood loss in menstruation and child birth. The direct effect of hemoglobin on mortality may only be measured in the absence of blood transfusions. While this study tries to deal with confounding effects of blood transfusions, it suggests that women do not survive lower Hb levels than men. Furthermore, women in this study received less blood transfusions than men with the same hemoglobin value (Supplementary Figure S1), putting women at an advantage in terms of transfusion driven morbidity. However, this could also mean that current intervention thresholds put women at a survival disadvantage.

This study underscores the strong association of low preoperative Hb levels with increased in-hospital mortality that has been demonstrated in previous studies (7, 24, 25). This study further highlights the potential of preoperative hemoglobin for perioperative risk assessment and emphasizes the importance of sex-specific consideration of hemoglobin thresholds. Similar to what has been shown for cardiac surgery patients (6), this study showed for a broader surgical population that low preoperative Hb increased transfusion requirements and prolonged hospital stay for patients.

It is important to acknowledge some limitations. In a surgical cohort even in the absence of comorbidities patients will have more adverse outcomes than a healthy sample of the population, which limits generalization. However, the higher number of adverse outcomes enables the outcome oriented approach of threshold estimation employed in this study. This study also lacks standardized transfusion thresholds, which could introduce red cell transfusions as a confounder between preoperative hemoglobin and outcomes of this study. This aspect could also be beneficial since patients from 14 different hospitals could capture a diverse clinical perspective of transfusion strategies in Germany. To minimize confounding by transfusion thresholds, models were evaluated that included red cell transfusions as a confounder. There are other potential confounders, which may contribute to mortality, that were not assessed in this study: intraoperative stress, bleeding and bleeding disorders, and reactions and negative direct effects caused by blood transfusions. While these confounders could not be directly assessed, the number of units of red cell, plasma, platelet, fibrinogen and prothrombin complex concentrate were evaluated. In all of the aforementioned cases the number of transfusions should capture most of the confounding effect as a mediator variable. Furthermore, a diverse surgical population was evaluated in this study, which might not fully capture effects in specific subpopulations. Our modelling approach sought to limit any bias introduced by this and a subgroup analysis for all surgical disciplines included in this study was conducted for the primary outcome. Lastly, this study focused on in-hospital mortality, which is a rather rare outcome and may limit power. In-hospital mortality can also be a biased measure and may not accurately capture true case mortality (26).

Further research is needed to examine the impact of preoperative Hb levels on other long-term outcomes, such as post-discharge mortality and morbidity. Future studies should also determine the validity and generalizability of these findings. Until such investigations are completed, it would be premature to alter current intervention protocols based on this research. Nonetheless, it is advisable to encourage healthcare professionals to monitor Hb disparities between males and females and to strive for the optimization of preoperative Hb levels.

In conclusion, this study confirmed the strong relationship between preoperative anemia and adverse outcomes and proposes Hb thresholds for survival equity between men and women. The findings contribute to ongoing efforts to establish evidence-based Hb thresholds and provide valuable insights for clinical decision-making in the diagnosis and management of anemia. By optimizing anemia treatment strategies and promoting equitable healthcare delivery, patient outcomes can be significantly improved.

## Data availability statement

The raw data supporting the conclusions of this article will be made available by the authors, without undue reservation. For original data, please contact: patientbloodmanagement@ukffm.de.

## Ethics statement

The studies involving humans were approved by Ethics committee of the University Hospital Frankfurt. The studies were conducted in accordance with the local legislation and institutional requirements. The ethics committee/institutional review board waived the requirement of written informed consent for participation from the participants or the participants’ legal guardians/next of kin because routine clinical data was collected.

## Author contributions

FR: Conceptualization, Data curation, Formal analysis, Investigation, Methodology, Visualization, Writing – original draft, Writing – review & editing. LH: Conceptualization, Data curation, Methodology, Project administration, Validation, Writing – review & editing. OO: Data curation, Methodology, Validation, Writing – review & editing. PF: Data curation, Resources, Writing – review & editing. JF: Data curation, Resources, Writing – review & editing. JT: Data curation, Resources, Writing – review & editing. MW: Data curation, Resources, Writing – review & editing. KZ: Funding acquisition, Project administration, Resources, Supervision, Writing – review & editing. SC: Conceptualization, Data curation, Investigation, Project administration, Supervision, Validation, Writing – review & editing. PM: Conceptualization, Funding acquisition, Investigation, Project administration, Resources, Supervision, Writing – review & editing.

## Group member of German Patient Blood Management Network Collaborators

Olaf Baumhove, Samuel de Leeuw van Weenen (Department of Anaesthesiology, Intensive Care Medicine and Pain Therapy), Klinikum Westmuensterland Bocholt; Markus Velten, Maria Wittmann, Claudia Neumann, Andrea Kirfel, Nadine Straßberger-Nerschbach, Heidi Ehrentraut, Daniel Grigutsch, Vera Guttenthaler, Alma Puskarevic, Ghaith Mohssen (Department of Anaesthesiology and Operative Intensive Care Medicine), Johannes Oldenburg (Institute of Experimental Haematology and Transfusion Medicine), Jan Görtzen (Department of Internal Medicine I), University Hospital Bonn; Diana Narita, Lighvani Barbara (Institute of Laboratory Diagnostics und Transfusion Medicine), Josef Michael Huber (Institute of Laboratory Diagnostics, Immunohematology and Microbiology), DONAUISAR Klinikum (Deggendorf & Dingolfing); Lea Valeska Blum, Suma Choorapoikayil, Lotta Hof, Sabine Isik, Vanessa Neef, Florian Piekarski, Kai Zacharowski (Department of Anaesthesiology, Intensive Care Medicine and Pain Therapy), Thomas Walther, Harald Keller (Department of Thoracic and Cardiovascular Surgery), Andreas Schnitzbauer (Department of General and Visceral Surgery), Thomas Schmitz-Rixen, Kyriakos Oikonomou (Department of Vascular and Endovascular Surgery), Bjoern Steffen (Department of Haematooncology), Stefan Zeuzem (Department of Gastroenterology and Hepatology), Marcus Czabanka (Department of Neurosurgery), Felix Chun (Department of Urology), Ingo Marzi, (Department of Trauma, Hand and Reconstructive Surgery), Timo Stöver (Department of Ear, Nose and Throat), Shahram Ghanaati (Department of Oral Maxillofacial and Plastic Facial Surgery), Frank Louwen, (Department of Gynecology and Obstetrics), University Hospital Frankfurt; Markus M. Mueller, Christoph Geisen, Erhard Seyfried (German Red Cross Blood Transfusion Service Baden-Wuerttemberg– Hessen, Institute of Transfusion Medicine and Immunohematology), Eva Herrmann (Institute of Biostatistics and Mathematical Modelling, Department of Medicine), University Frankfurt; Alexandra Bayer (Department of Anaesthesiology and Intensive Care Medicine), Agatharied Hospital Hausham; Henry Weigt, Björn Lange (Department of Anaesthesiology), SLK-Kliniken Heilbronn; Ansgar Raadts (Department of Anaesthesiology and Intensive Care Medicine), Christoph Haas (Executive Department for Structure, Process and Quality Management), University Hospital Jena; Johannes Duemmler (Department of Anaesthesiology and Intensive Care), Ulf Lorenzen (Department of Anaesthesiology and Intensive Care), Matthias Pagel (Information-Technology UKSH), Thomas Puehler (Department of Cardiovascular Surgery), Julius Pochhammer (Department of General and Visceral Surgery), Tim Klueter (Department of Trauma, Hand and Reconstructive Surgery), Hajrullah Ahmeti (Department of Neurosurgery), Dirk Bauerschlag (Department of Gynaecology and Obstetrics), Henning Wieker (Department of Oral Maxillofacial and Plastic Facial Surgery), René Rusch (Department of Vascular Surgery), University Medical Center Schleswig-Holstein; Jens Friedrich, Gerd Molter (Department of Anaesthesiology and Intensive Care Medicine), Klinikum Leverkusen; Brigitte Bräutigam (Business Unit Hospital Controlling) Patrick Friederich (Department of Anaesthesiology, Critical Care Medicine, Pain Therapy), München Klinik (Bogenhausen); Dana Janina Jenke, Kira Kieserling, Dennie Scholle, Andrea U. Steinbicker, Alexander Zarbock (Department of Anaesthesiology, Intensive Care Medicine and Pain Therapy), Sven Martens (Department of Thoracic and Cardiovascular Surgery), Andres Schrader (Clinic for Urology and Pediatric Urology), R. G. Geissler, H. Hillmann (Institute of Transfusion Medicine), Georg Lenz (Medical Clinic A, Haematology, Pneumology, Haemostaseology and Oncology), University Hospital Muenster; Klaus Schwendner (Department of Anaesthesiology and Operative Intensive Care Medicine), Diakonie Hospital Nuremberg; Lukas Spitzmüller, Josef Thoma, Viola Weber (Department of Anesthesiology and Operative Intensive Care Medicine), Ortenau Klinikum Offenburg-Gengenbach/Kehl; Philipp Helmer, Sebastian Hottenrott, Peter Kranke, Patrick Meybohm, Daniel Roeder, Tobias Schlesinger, Magdalena Sitter, Jan Stumpner (Department of Anaesthesiology, Intensive Care, Emergency and Pain Medicine), University Hospital Wuerzburg.
