# Supplementary material for: Preoperative hemoglobin thresholds for survival equity in women and men
Source: Front Med (Lausanne). 2024 Mar 13;11:1334773. doi: 10.3389/fmed.2024.1334773 (PMC10965651; doi:10.3389/fmed.2024.1334773)
Supplement: Supplementary file 1 [file Table_1.docx]

**Supplementary table 1**: OPS codes classified as major surgery

| Discipline | OPS |
| --- | --- |
| Visceral surgery | 5-454.10, 5-454.20, 5-454.21, 5-454.60, 5-454.x, 5-455.01, 5-455.25, 5-455.41, 5-455.42, 5-455.45, 5-455.5x, 5-455.61, 5-455.71, 5-455.72, 5-455.75, 5-455.91, 5-455.95, 5-456.00, 5-460.50, 5-460.51, 5-461.21, 5-461.31, 5-463.10, 5-463.20, 5-465.1, 5-465.2, 5-466.2, 5-467.02, 5-469.00, 5-469.10, 5-469.11, 5-469.20, 5-469.21, 5-469.70, 5-469.71, 5-469.80, 5-469.xx, 5-482.80, 5-484.31, 5-484.32, 5-484.35, 5-484.52, 5-484.55, 5-484.59, 5-485.01, 5-486.1, 5-486.4, 5-501.00, 5-501.01, 5-501.20, 5-501.21, 5-501.23, 5-502.0, 5-502.1, 5-502.2, 5-502.4, 5-502.5, 5-502.6, 5-504.0, 5-504.1, 5-505.0, 5-505.1, 5-524.00, 5-524.1, 5-524.2, 5-525.0, 5-525.1, 5-554.41, 5-554.71, 5-554.83, 5-555.0, 5-685.00, 5-431.0, 5-431.2x, 5-433.0, 5-435.1, 5-437.23, 5-437.25, 5-437.x3, 5-445.10, 5-445.20, 5-448.62, 5-449.x0, 5-450.1, 5-340.0, 5-383.9g, 5-413.11, 5-424.12, 5-426.02, 5-426.21 |
| Vascular surgery | 5-380.12, 5-380.53, 5-380.54, 5-380.56, 5-380.70, 5-380.71, 5-380.72, 5-380.73, 5-380.80, 5-380.87, 5-380.a2, 5-381.00, 5-381.01, 5-381.02, 5-381.33, 5-381.54, 5-381.70, 5-381.71, 5-381.72, 5-381.83, 5-382.33, 5-383.64, 5-383.70, 5-383.84, 5-384.42, 5-384.46, 5-384.61, 5-384.66, 5-384.72, 5-384.74, 5-384.76, 5-388.24, 5-388.54, 5-388.70, 5-388.71, 5-388.9b, 5-38a.41, 5-38a.70, 5-38a.80, 5-38a.8c, 5-38a.8f, 5-38a.b1, 5-38a.c0, 5-38a.x, 5-393.02, 5-393.2, 5-393.36, 5-393.42, 5-393.49, 5-393.51, 5-393.52, 5-393.53, 5-393.54, 5-393.55, 5-393.61, 5-393.8, 5-394.1, 5-394.2, 5-394.4, 5-394.5, 5-396.40, 5-413.10, 5-454.20, 5-864.4, 5-864.5, 5-864.9, 5-864.a |
| Urology | 5-388.97, 5-553.00, 5-553.01, 5-553.03, 5-554.40, 5-554.41, 5-554.43, 5-554.50, 5-554.a0, 5-554.a2, 5-554.a3, 5-554.b0, 5-557.40, 5-557.41, 5-557.43, 5-575.x0, 5-576.60, 5-576.70 |
| Thoracic and cardiac surgery | 5-322.d1, 5-322.d2, 5-322.d3, 5-322.g1, 5-324.31, 5-324.b1, 5-324.b2, 5-325.11, 5-331, 5-333.1, 5-340.1, 5-340.a, 5-340.b, 5-340.c, 5-340.d, 5-343.0, 5-344.13, 5-344.3, 5-344.40, 5-345.4, 5-345.5, 5-346.4, 5-346.81, 5-349.1, 5-349.3, 5-351.02, 5-351.04, 5-351.05, 5-351.06, 5-351.12, 5-351.14, 5-351.22, 5-351.24, 5-351.42, 5-352.01, 5-352.03, 5-352.05, 5-352.10, 5-352.11, 5-352.13, 5-353.0, 5-353.1, 5-353.2, 5-353.4, 5-353.6, 5-354.01, 5-354.03, 5-354.08, 5-354.09, 5-354.0a, 5-354.11, 5-354.12, 5-354.13, 5-354.31, 5-356.2, 5-357.2, 5-358.02, 5-358.03, 5-358.04, 5-358.05, 5-35a.00, 5-35a.01, 5-35a.03, 5-35a.04, 5-35a.30, 5-35a.31, 5-35a.41, 5-35a.5, 5-35a.60, 5-361.03, 5-361.07, 5-361.13, 5-361.17, 5-361.23, 5-361.27, 5-361.33, 5-362.03, 5-362.07, 5-362.13, 5-362.33, 5-362.37, 5-362.63, 5-362.93, 5-363.x, 5-369.0, 5-370.0, 5-370.3, 5-372.2, 5-373.0, 5-373.1, 5-373.4, 5-373.x, 5-374.3, 5-374.6, 5-376.40, 5-376.41, 5-378.52, 5-378.55, 5-378.5c, 5-378.5f, 5-378.61, 5-378.75, 5-379.1, 5-379.5, 5-37b.00, 5-37b.30, 5-380.31, 5-384.01, 5-384.02, 5-384.8, 5-388.30, 5-388.31, 5-38a.70, 5-395.32 |
| Trauma and orthopaedic surgery | 5-012.0, 5-312.0, 5-37b.20, 5-413.10, 5-790.4e, 5-790.4f, 5-790.5f, 5-790.6d, 5-790.8e, 5-791.kg, 5-792.3g, 5-792.kg, 5-793.3f, 5-793.5e, 5-793.kf, 5-794.2f, 5-794.4e, 5-794.ae, 5-794.af, 5-798.2, 5-798.3, 5-798.4, 5-798.5, 5-799.3, 5-799.4, 5-800.2g, 5-800.3g, 5-800.70, 5-820.00, 5-820.01, 5-820.02, 5-820.21, 5-820.30, 5-820.41, 5-820.50, 5-821.15, 5-821.24, 5-821.33, 5-821.43, 5-821.63, 5-821.7, 5-821.f4, 5-822.81, 5-822.92, 5-822.g1, 5-823.27, 5-823.2b, 5-823.42, 5-824.20, 5-824.21, 5-825.00, 5-825.12, 5-825.k1, 5-825.kx, 5-829.f, 5-829.h, 5-829.k0, 5-829.k1, 5-829.k3, 5-829.r, 5-830.0, 5-831.0, 5-836.30, 5-836.31, 5-836.32, 5-836.33, 5-836.50, 5-839.0, 5-839.a0, 5-839.a1, 5-839.a2, 5-839.a3, 5-83b.20, 5-83b.21, 5-83b.22, 5-83b.30, 5-83b.31, 5-83b.32, 5-83b.41, 5-83b.42, 5-83b.43, 5-83b.51, 5-83b.52, 5-83b.53, 5-83w.0, 5-83w.1, 5-83w.20, 5-862.4, 5-864.8, 5-864.a |
| Neurosurgery | 5-010.00, 5-010.2, 5-011.2, 5-012.0, 5-012.1, 5-012.3, 5-012.x, 5-015.0, 5-015.1, 5-015.20, 5-015.21, 5-015.3, 5-015.4, 5-015.x, 5-020.1x, 5-020.2, 5-020.70, 5-020.72, 5-022.00, 5-022.01, 5-022.10, 5-022.13, 5-025.2, 5-025.3, 5-025.4, 5-025.7, 5-025.x, 5-026.1, 5-311.0, 5-311.1, 5-832.4, 5-832.5, 5-836.53, 5-837.00, 5-837.01, 5-839.5, 5-839.60, 5-839.61, 5-839.62, 5-839.a0, 5-839.a1, 5-83b.20, 5-83b.31, 5-83b.50, 5-83b.51, 5-83b.52, 5-83b.53, 5-83b.70, 5-83b.71 |
| Oral-maxillofacial surgery | 5-020.68, 5-231.03, 5-231.10, 5-231.11, 5-231.13, 5-231.33, 5-231.x3, 5-241.0, 5-244.30, 5-251.00, 5-251.02, 5-251.0x, 5-251.x0, 5-252.32, 5-252.42, 5-277.02, 5-277.20, 5-277.x0, 5-277.xx, 5-278.00, 5-278.02, 5-278.22, 5-278.34, 5-279.0 |
| Gynecology | 5-455.04, 5-455.71, 5-469.21, 5-484.51, 5-502.0, 5-681.90, 5-681.92, 5-681.94, 5-682.01, 5-682.02, 5-683.00, 5-683.01, 5-683.02, 5-683.03, 5-683.04, 5-683.10, 5-683.11, 5-683.12, 5-683.13, 5-683.14, 5-683.20, 5-683.22, 5-683.23, 5-683.3, 5-683.7, 5-685.00, 5-685.02, 5-685.1, 5-685.3, 5-685.41, 5-685.43, 5-687.0, 5-687.2, 5-695.00, 5-695.02, 5-695.10 |
