# Supplementary material for: Preoperative hemoglobin thresholds for survival equity in women and men
Source: Front Med (Lausanne). 2024 Mar 13;11:1334773. doi: 10.3389/fmed.2024.1334773 (PMC10965651; doi:10.3389/fmed.2024.1334773)
Supplement: Supplementary file 2 [file Table_2.docx]

**Supplementary table 2**: Mortality in men and women at different levels of preoperative hemoglobin

| Preoperative hemoglobin (g/dl) | Men | | Women | | p |
| --- | --- | --- | --- | --- | --- |
|  | n | Deaths | n | Deaths |  |
| 4.0-6.9 | 2,923 | 379 (12.97%) | 2,368 | 260 (10.98%) | 0.3 |
| 7.0-7.9 | 4,487 | 527 (11.75%) | 3,722 | 359 (9.65%) | 0.025 |
| 8.0-8.9 | 9,409 | 1,017 (10.81%) | 8,072 | 738 (9.14%) | 0.0028 |
| 9.0-9.9 | 16,085 | 1,427 (8.87%) | 15,810 | 1,098 (6.94%) | <0.001 |
| 10.0-10.9 | 23,834 | 1,575 (6.61%) | 27,097 | 1,226 (4.52%) | <0.001 |
| 11.0-11.9 | 34,534 | 1,739 (5.04%) | 47,579 | 1,309 (2.75%) | <0.001 |
| 12.0-12.9 | 50,309 | 1,629 (3.24%) | 83,213 | 1,283 (1.54%) | <0.001 |
| 13.0-13.9 | 77,051 | 1,510 (1.96%) | 107,254 | 1,016 (0.95%) | <0.001 |
| 14.0-14.9 | 105,134 | 1,177 (1.12%) | 67,800 | 562 (0.83%) | <0.001 |
| 15.0-15.9 | 87,319 | 715 (0.82%) | 18,911 | 242 (1.28%) | <0.001 |
| 16.0-18.0 | 45,271 | 454 (1%) | 3,948 | 128 (3.24%) | <0.001 |
