# Supplementary material for: Preoperative hemoglobin thresholds for survival equity in women and men
Source: Front Med (Lausanne). 2024 Mar 13;11:1334773. doi: 10.3389/fmed.2024.1334773 (PMC10965651; doi:10.3389/fmed.2024.1334773)
Supplement: Supplementary file 3 [file Table_3.docx]

**Supplementary table 3**: Fixed effects on in-hospital mortality from generalized additive model

| Variable | Estimate | Std. Error | p |
| --- | --- | --- | --- |
| Intercept | -2.1 | 0.14 | <0.001 |
| Demographic risk | 0.094 | 0.0014 | <0.001 |
| Surgical discipline |  |  |  |
| Cardiac surgery | -0.72 | 0.1 | <0.001 |
| Dermatology | -0.92 | 0.11 | <0.001 |
| Endocrine surgery | -2.6 | 0.39 | <0.001 |
| Gynaecology | -3.2 | 0.23 | <0.001 |
| Neurosurgery | 0.17 | 0.099 | 0.084 |
| Ophthalmology | -3.1 | 0.2 | <0.001 |
| Oral-maxillofacial surgery | -1.8 | 0.14 | <0.001 |
| Otorhinolaryngology | 0.2 | 0.1 | 0.045 |
| Surgery of the hematopoietic- and lymphatic system | -1.7 | 0.13 | <0.001 |
| Thoracic surgery | -0.0024 | 0.1 | 0.981 |
| Trauma and orthopaedic surgery | -0.89 | 0.099 | <0.001 |
| Urology | -1.3 | 0.1 | <0.001 |
| Vascular surgery | -0.18 | 0.098 | 0.072 |
| Visceral surgery | -0.16 | 0.096 | 0.103 |
| Major surgery | 0.78 | 0.019 | <0.001 |
| Charlson comorbidity index | 0.17 | 0.0026 | <0.001 |
| Follow-up (days) | -0.011 | 0.0005 | <0.001 |
| Female | -0.097 | 0.016 | <0.001 |
| Red blood cell units | 0.13 | 0.0014 | <0.001 |
| Preoperative hemoglobin (1 g/dl decrease) | 0.19 | 0.0035 | <0.001 |
