# Supplementary material for: Preoperative hemoglobin thresholds for survival equity in women and men
Source: Front Med (Lausanne). 2024 Mar 13;11:1334773. doi: 10.3389/fmed.2024.1334773 (PMC10965651; doi:10.3389/fmed.2024.1334773)
Supplement: Supplementary file 4 [file Table_4.docx]

**Supplementary table 4**: German Patient Blood Management Network Collaborators

| Center | Collaborators |
| --- | --- |
| Klinikum Westmuensterland Bocholt | Olaf Baumhove, Samuel de Leeuw van Weenen (Department of Anaesthesiology, Intensive Care Medicine and Pain Therapy). |
| University Hospital Bonn | Markus Velten, Maria Wittmann, Claudia Neumann, Andrea Kirfel, Nadine Straßberger-Nerschbach, Heidi Ehrentraut, Daniel Grigutsch, Vera Guttenthaler, Alma Puskarevic, Ghaith Mohssen (Department of Anaesthesiology and Operative Intensive Care Medicine), Johannes Oldenburg (Institute of Experimental Haematology and Transfusion Medicine), Jan Görtzen (Department of Internal Medicine I). |
| DONAUISAR Klinikum (Deggendorf & Dingolfing) | Diana Narita, Lighvani Barbara (Institute of Laboratory Diagnostics und Transfusion Medicine), Josef Michael Huber (Institute of Laboratory Diagnostics, Immunohematology and Microbiology). |
| University Hospital Frankfurt | Lea Valeska Blum, Suma Choorapoikayil, Lotta Hof, Sabine Isik, Vanessa Neef, Florian Piekarski, Kai Zacharowski (Department of Anaesthesiology, Intensive Care Medicine and Pain Therapy), Thomas Walther, Harald Keller (Department of Thoracic and Cardiovascular Surgery), Andreas Schnitzbauer (Department of General and Visceral Surgery), Thomas Schmitz-Rixen, Kyriakos Oikonomou (Department of Vascular and Endovascular Surgery), Bjoern Steffen (Department of Haematooncology), Stefan Zeuzem (Department of Gastroenterology and Hepatology), Marcus Czabanka (Department of Neurosurgery), Felix Chun (Department of Urology), Ingo Marzi,  (Department of Trauma, Hand and Reconstructive Surgery), Timo Stöver (Department of Ear, Nose and Throat), Shahram Ghanaati (Department of Oral Maxillofacial and Plastic Facial Surgery), Frank Louwen, (Department of Gynecology and Obstetrics). |
| University Frankfurt | Markus M. Mueller, Christoph Geisen, Erhard Seyfried (German Red Cross Blood Transfusion Service Baden-Wuerttemberg– Hessen, Institute of Transfusion Medicine and Immunohematology), Eva Herrmann (Institute of Biostatistics and Mathematical Modelling, Department of Medicine). |
| Agatharied Hospital Hausham | Alexandra Bayer (Department of Anaesthesiology and Intensive Care Medicine). |
| SLK-Kliniken Heilbronn | Henry Weigt, Björn Lange (Department of Anaesthesiology). |
| University Hospital Jena | Ansgar Raadts (Department of Anaesthesiology and Intensive Care Medicine), Christoph Haas (Executive Department for Structure, Process and Quality Management). |
| University Medical Center Schleswig-Holstein | Johannes Duemmler (Department of Anaesthesiology and Intensive Care), Ulf Lorenzen (Department of Anaesthesiology and Intensive Care), Matthias Pagel (Information-Technology UKSH), Thomas Puehler (Department of Cardiovascular Surgery), Julius Pochhammer (Department of General and Visceral Surgery), Tim Klueter (Department of Trauma, Hand and Reconstructive Surgery), Hajrullah Ahmeti (Department of Neurosurgery), Dirk Bauerschlag (Department of Gynaecology and Obstetrics), Henning Wieker (Department of Oral Maxillofacial and Plastic Facial Surgery), René Rusch (Department of Vascular Surgery). |
| Klinikum Leverkusen | Jens Friedrich, Gerd Molter (Department of Anaesthesiology and Intensive Care Medicine). |
| München Klinik (Bogenhausen) | Brigitte Bräutigam (Business Unit Hospital Controlling) Patrick Friederich (Department of Anaesthesiology, Critical Care Medicine, Pain Therapy). |
| University Hospital Muenster | Dana Janina Jenke, Kira Kieserling, Dennie Scholle, Andrea U. Steinbicker, Alexander Zarbock (Department of Anaesthesiology, Intensive Care Medicine and Pain Therapy), Sven Martens (Department of Thoracic and Cardiovascular Surgery), Andres Schrader (Clinic for Urology and Pediatric Urology), R. G. Geissler, H. Hillmann (Institute of Transfusion Medicine), Georg Lenz (Medical Clinic A, Haematology, Pneumology, Haemostaseology and Oncology). |
| Diakonie Hospital Nuremberg | Klaus Schwendner (Department of Anaesthesiology and Operative Intensive Care Medicine). |
| Ortenau Klinikum Offenburg-Gengenbach/Kehl | Lukas Spitzmüller, Josef Thoma, Viola Weber (Department of Anesthesiology and Operative Intensive Care Medicine). |
| University Hospital Wuerzburg | Philipp Helmer, Sebastian Hottenrott, Peter Kranke, Patrick Meybohm, Daniel Roeder, Tobias Schlesinger, Magdalena Sitter, Jan Stumpner (Department of Anaesthesiology, Intensive Care, Emergency and Pain Medicine). |
