# Supplementary figures and images for: Preoperative hemoglobin thresholds for survival equity in women and men
Source: Front Med (Lausanne). 2024 Mar 13;11:1334773. doi: 10.3389/fmed.2024.1334773 (PMC10965651; doi:10.3389/fmed.2024.1334773)

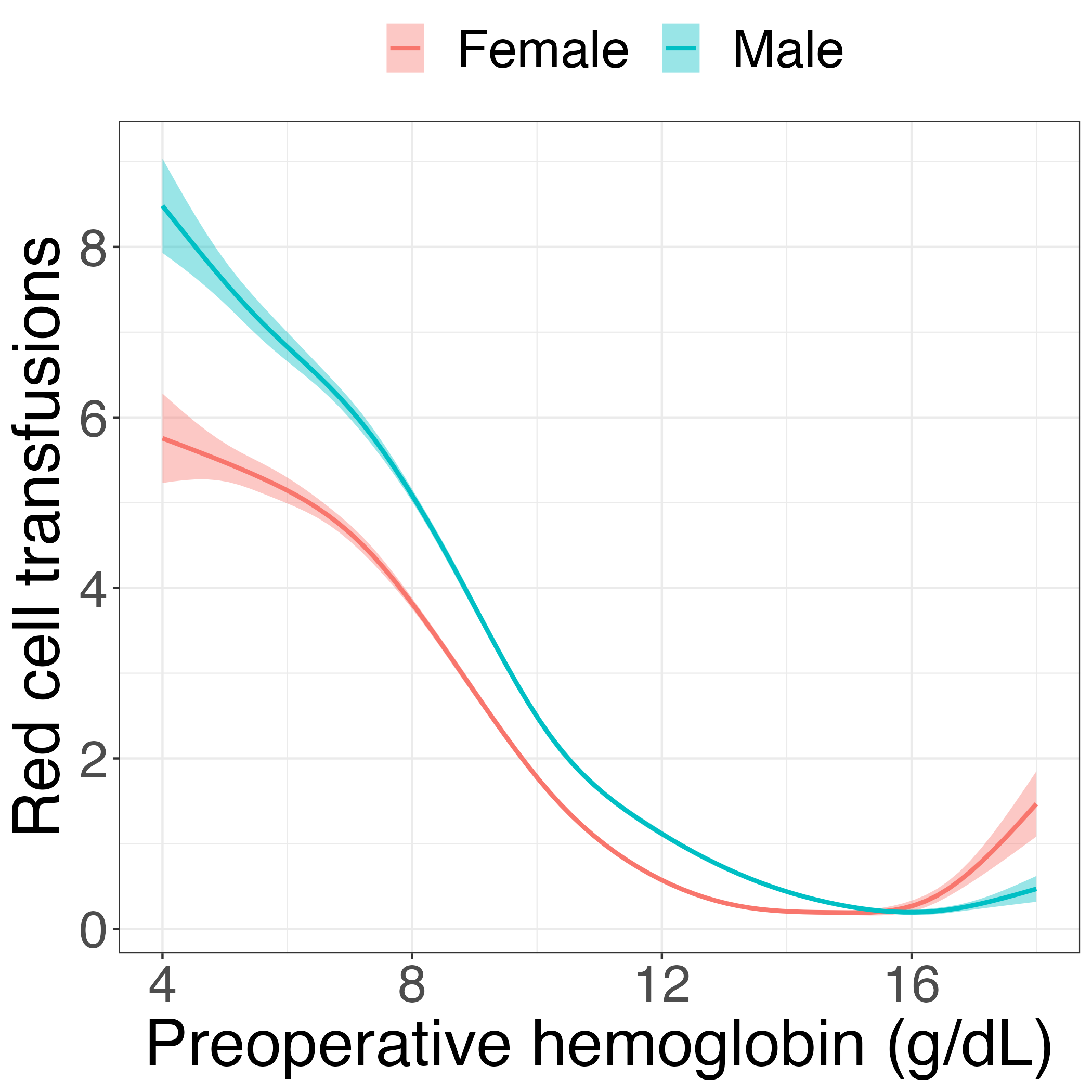

Supplement: Supplementary file 5 [file Image_1.png]
